# The Androgen Receptor and Its Crosstalk With the Src Kinase During Castrate-Resistant Prostate Cancer Progression

**DOI:** 10.3389/fonc.2022.905398

**Published:** 2022-06-27

**Authors:** Lin Gao, Bo Han, Xuesen Dong

**Affiliations:** ^1^ Department of Urologic Sciences, Faculty of Medicine, University of British Columbia, Vancouver, BC, Canada; ^2^ The Key Laboratory of Experimental Teratology, Ministry of Education and Department of Pathology, School of Basic Medical Sciences, Cheeloo College of Medicine, Shandong University, Jinan, China

**Keywords:** prostate cancer, Src kinase, androgen receptor, castrate-resistant prostate cancer, UGT2B17

## Abstract

While the androgen receptor (AR) signalling is the mainstay therapeutic target for metastatic prostate cancers, these tumours will inevitably develop therapy resistance to AR pathway inhibitors suggesting that prostate tumour cells possess the capability to develop mechanisms to bypass their dependency on androgens and/or AR to survive and progress. In many studies, protein kinases such as Src are reported to promote prostate tumour progression. Specifically, the pro-oncogene tyrosine Src kinase regulates prostate cancer cell proliferation, adhesion, invasion, and metastasis. Not only can Src be activated under androgen depletion, low androgen, and supraphysiological androgen conditions, but also through crosstalk with other oncogenic pathways. Reciprocal activations between Src and AR proteins had also been reported. These findings rationalize Src inhibitors to be used to treat castrate-resistant prostate tumours. Although several Src inhibitors had advanced to clinical trials, the failure to observe patient benefits from these studies suggests that further evaluation of the roles of Src in prostate tumours is required. Here, we summarize the interplay between Src and AR signalling during castrate-resistant prostate cancer progression to provide insights on possible approaches to treat prostate cancer patients.

## Introduction

Androgens and the androgen receptor (AR) play key roles during the development of prostate cancer (PCa). They are also tightly coupled with PCa progression to the castrate-resistant prostate cancer (CRPC) state. Targeting AR signalling has been a mainstay therapeutic option to manage locally advanced and metastatic PCa (1, 2). These therapies include inhibitors of androgen synthesis through the hypothalamus-pituitary-adrenal axis (e.g., leuprorelin) (3, 4), AR antagonist that prevent androgens from binding to AR (e.g., bicalutamide) (5), and more potent new generation AR inhibitors (e.g., abiraterone acetate and enzalutamide) (6, 7). Despite the maximal androgen ablation therapies that could be possibly applied to patients, therapy-resistant tumours are inevitably developed (6, 7). While the majority of CRPC remains AR-positive and presents an adenocarcinoma phenotype, more potent AR pathway inhibitor (ARPI) treatment often induced AR negative neuroendocrine prostate cancer (NEPC) or double-negative prostate tumours (DNPC) accounting for ~20% of CRPC (8–11). These findings indicate that the phenotypes of CRPC tumour cells are heterogeneous and are associated with the potency and duration of ARPI treatments. AR target therapies trigger various cellular mechanisms that promote tumour evolution to bypass the dependency of androgens and/or AR. Supporting this notion, PCa patients can benefit from intermittent androgen deprivation therapy (1) and bipolar androgen therapy (12–14) that either recover endogenous androgens or supplement exogenous androgens to delay disease progression. Therefore, CRPC is not an initial clinical presentation of prostate cancer, but the consequence of anti-AR therapies.

## Castrate-Resistant Prostate Cancer Progression Is Coupled With AR Signalling

Except for small cell carcinoma and DNPC, most CRPC tumours express AR proteins (8–11). The questions remain whether an AR-positive CRPC tumour is driven by the AR signalling, or it is AR indifferent and other oncogenic pathways replace AR to promote tumour progression. If the AR drives the progression of the CRPC tumour, does the AR rely on the castrate levels of androgens or act in a ligand-independent manner? It is important to differentiate tumours by various modes of action of AR so that effective ARPI treatments could be used.

**Figure 1:**
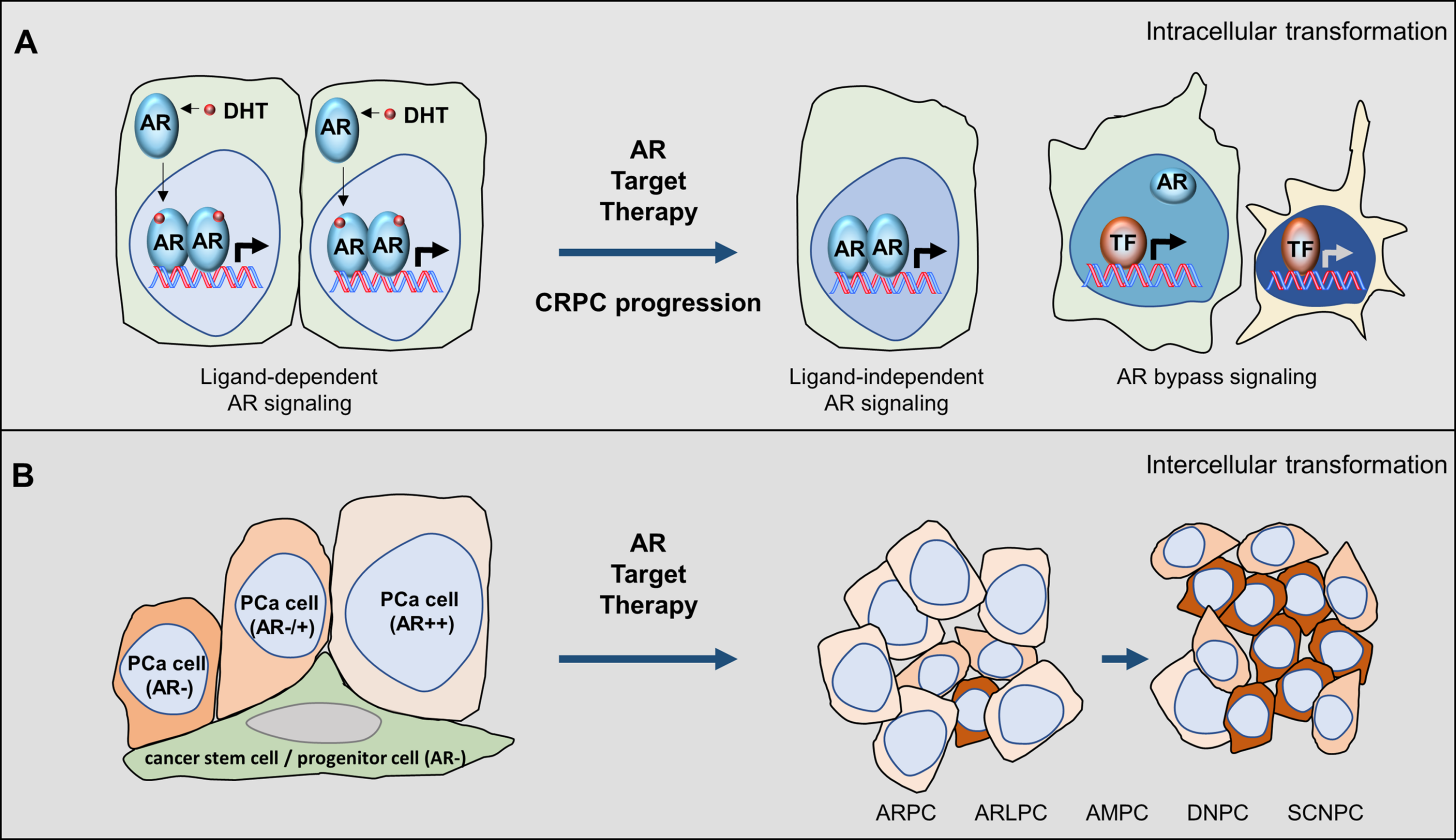
Castrate-resistant prostate cancer progression is coupled with AR pathway inhibitions. **(A)** Intracellular mechanisms can be adopted by PCa cells under ARPI treatments that shift the ligand-dependent AR signalling to the ligand-independent AR signalling and AR bypass oncogenic pathways. **(B)** Intercellular mechanisms can be adopted by tumours under ARPI treatments to select tumours that are less dependent on androgens to survive and populate. During this process, tumours exhibit heterogeneous phenotypes that can be classified by luminal epithelial markers such as AR and PSA and neuroendocrine markers such as chromogranin.

Studies using *in vitro* cell models indicated that the ligand-dependent and ligand-independent AR signalling, and AR bypass mechanisms can all be possibly adopted by cancer cells. The LNCaP cell model, androgen-dependent tumour, can form xenografts only in non-castrated mice (15). These tumours are initially responsive to castration surgery but will progress into castrate-resistant xenografts (16). The CRPC LNCaP tumours are still responsive to ARPIs such as enzalutamide, emphasizing that these tumours still rely on ligand-dependent AR signalling (17). In contrast, the LNCaP95 cell model was derived from LNCaP cells but cultured under prolonged androgen deprivation conditions (18). These cells are naturally resistant to enzalutamide *in vitro* and *in vivo*, and can only be inoculated in castrated mice to form xenografts (19, 20). LNCaP95 xenografts are androgen-independent tumours, expressing high levels of AR, AR splice variants (AR-Vs), and AR target genes such as PSA. AR gene disruption by CRISPR demolishes the expression of AR and AR-V7 and reduces LNCaP95 cell growth, supporting that AR-mediated signalling is still functional to promote tumour progression (20, 21). However, the establishment of LNCaP95 cell clones with complete disruption of AR and AR-V protein expression indicates that the AR and AR-Vs do not necessitate the viability of LNCaP95 cells, highlighting the existence of AR bypass mechanisms. It had been shown that signalling mediated by glucocorticoid receptors may replace AR to promote CRPC progression (22). In PTEN deficient cancer cells, there exists a reciprocal activation between the AR and PI3K/Akt signalling. Inhibition of one will activate the other to maintain tumour cell survival (23, 24). Since genomic alterations of the PTEN gene and genes associated with the PI3K/Akt signalling are common in CRPC, this AR bypass mechanism is frequently used by PCa to counteract AR target therapy. These model systems indicate that PCa cells possess phenotypical plasticity to adapt to ARPI treatments by using various intracellular mechanisms to switch off the ligand-dependent AR signalling and switch on the ligand-independent AR signalling, and bypass AR through other oncogenic kinase pathways (**Figure 1**).

Based on these findings from cell models, we can speculate that AR target therapy will only provide short-term tumour suppression. Prolonged ARPI treatment will eventually transform the LNCaP type of tumours into the LNCaP95 type of tumours. Even though drugs can be successfully developed to degrade AR and AR splice variant proteins, LNCaP95 cells with complete AR gene destruction by CRISPR sets up an example that AR is not essential for PCa cell viability after prolonged AR target therapy. This hypothesis is supported by that small cell carcinoma, DNPC, and even AR indifferent tumours become more prevalent in patients treated with new generation ARPIs. Nevertheless, there still exists a therapeutic window for AR target therapy during the early stage of tumour progression.

## Heterogeneity of Castrate-Resistant Prostate Cancer

Tumour heterogeneity also complicates CRPC progression. The epithelium in the adult prostate contains luminal epithelial cells, neuroendocrine (NE) cells, and basal cells that are believed to be sustained in a homeostasis state by stem/progenitor cells within the basal layer through differentiation (25). The AR is expressed in luminal epithelial cells when they are terminally differentiated from progenitor cells (26), and acts to maintain the differentiation but does not regulate cell proliferation (27). While PCa is predominantly derived from luminal epithelial cells, the AR signal in PCa cells can also promote PCa cell proliferation and metastasis (28, 29). ARPIs would thereby not only inhibit cell growth but also trigger PCa cells to de-differentiate and gain lineage plasticity (30). Because heterogeneous cell populations co-exist in PCa tissues, ARPIs can block the androgen-sensitive cells from growing and simultaneously select androgen-insensitive cells to populate. These surviving cells may also contain cells that are derived from prostate progenitor cells but have not differentiated to the luminal epithelial lineage. There are at least five different CRPC tumours reported in patients that can be classified based on AR signature and NE biomarkers: 1) AR-high tumours (ARPC) containing cancer cells expressing uniform AR and AR target genes and no NE markers; 2) AR-low tumours (ARLPC) contains cells with weak or heterogenous AR and PSA expression with no NE markers; 3) amphicrine PCa (AMPC) containing cells co-expressing AR, PSA, and NE markers; 4) small cell neuroendocrine PCa (SCNPC) containing cells with classic small cell carcinoma histology and NE marker positive and no AR and PSA expression; and 5) DNPC containing cells with neither NE marker and AR expression (8, 9). These observations are recapitulated in patient-derived xenografts (8, 9, 31–34). Since treatment naïve PCa is predominantly AR and PSA positive tumours, the presentation of ARLPC, SCNPC, and DNPC at the late stage of tumour progression suggests that AR bypass mechanisms are adopted by these tumours.

The mechanisms by which the transition of AR high PCa to AR negative SCNPC or DNPC are not fully defined. Two possible mechanisms have been proposed (**Figure 1A**). The co-expression of AR signalling and NE biomarkers in the same cancer cells supports a “trans-differentiation” mechanism by which existing AR-positive PCa cells undergo de-differentiation by ARPIs and gain an NE lineage. It had been shown that androgen deprivation, chemotherapy reagents, UV light exposure, cytokines as well as hypoxia can all transiently induce NE differentiation of androgen-sensitive PCa cells (35–40). The other possibility is the “clonal selection” mechanism by which the prostate stem-like/progenitor cells gain oncogenic capacities and differentiate into basal, stemness, luminal, and NE lineages (**Figure 1B**). These progenitor cells are AR negative and have a low proliferation index, thereby, are resistant to ARPIs and/or systematic radiation and chemotherapy. The androgen-sensitive LNCaP model was also reported to gain stem cell and neural phenotypes and tumour-initiating potential, in an androgen-free neural/neural crest (N/NC) stem medium (41). These findings indicate that PCa cells have an intrinsic capability to switch on differentiation to a different lineage under ARPI treatments.

In summary, the heterogeneity of prostate tumours is a natural challenge to AR target therapy. CRPC tumour cells have different genetic and epigenetic backgrounds, cell lineages, or evolutional stages (from being androgen-dependent to AR indifferent) within a tumour. Targeting AR signalling in the long-term is predictably ineffective since it blocks one cancer cell population and permits others to expand. Combinational therapies would be a future therapeutic option.

## Ligand-Independent AR Signalling in Prostate Cancer Cells

AR can exert its transcriptional activity in the absence of androgens through its N-terminal domain (NTD) (42). Androgen binding to AR triggers intramolecular interaction between NTD and the ligand-binding domain (LBD) that is required for ligand-dependent AR activity (43, 44). However, deletion of the LBD renders AR constitutively active (45). Endogenously expressed AR-Vs with truncated LBD have been demonstrated to mediate ligand-independent AR signalling to drive CRPC progression (46–48). AR-Vs are generated by the RNA splicing process, which is tightly coupled with AR gene transcription initiation and elongation rates (49). ARPIs increase the transcription rate of the AR gene (50) resulting in more AR pre-mRNA copies processed into mRNAs of either AR or AR-Vs by the RNA splicing machinery (49, 51). Therefore, when the LBD is truncated or blocked pharmacologically by ARPIs, it provides an opportunity for NTD to enable AR activation ligand independently.

The NTD is a domain with a flexible conformation that can interact with various coregulators to activate AR. It possesses the Tau5 domain (aa360-485) that mediates the ligand-independent activation of AR (52). Interestingly, the Src kinase uses its Src homology domain 3 (SH3) domain to recognize a polyproline motif (aa371-381) within AR and phosphorylates the Tyr534, which is required for AR activation by growth factors under androgen-depleted conditions (53). AR Tyr534 phosphorylation by Src also enhances AR protein stability through a mechanism that prevents AR from being recognized by the chaperone-associated ubiquitin ligase COOH terminus of Hsp70-interacting protein (CHIP) for proteasomal degradation (54).

Blocking the LBD by ARPIs creates opportunities for NTD to interact with growth factors, cytokines, and intracellular kinases to activate AR signalling. Epidermal growth factor (EGF) acts through its membrane receptor to stimulate Src activation, which in turn activates AR Tyr534 phosphorylation (53). AR phosphorylation at Tyr267 and Tyr363 can also be stimulated through Cdc42-associated kinase Ack by HER2 activation (55). IL-6 enhances the transcriptional activity of the AR NTD through STAT3, which action is associated with IL-6 stimulated MAPK/ERK pathways (56). The activation of the MAPK/ERK kinase pathways can enhance ligand-independent AR actions through modulating coactivators such as steroid receptor coactivator-1 (57). Furthermore, IL-8 can also stimulate ligand-independent activation of AR through the FAK and Src pathways (58). Given these growth factors and tumour-promoting cytokines are enriched in the tumour microenvironment, it is reasonable to believe that AR target therapy provides an opportunity for NTD to mediate ligand-independent AR signalling through crosstalk with intracellular kinases to confer tumours therapy resistance. Furthermore, activated kinases (e.g. Src) can trigger its downstream signal pathways to promote PCa cell survival, proliferation, and metastasis that may eventually bypass the requirement of the AR for tumour progression as we discuss later.

## The Src Tyrosine Kinases

**Figure 2:**
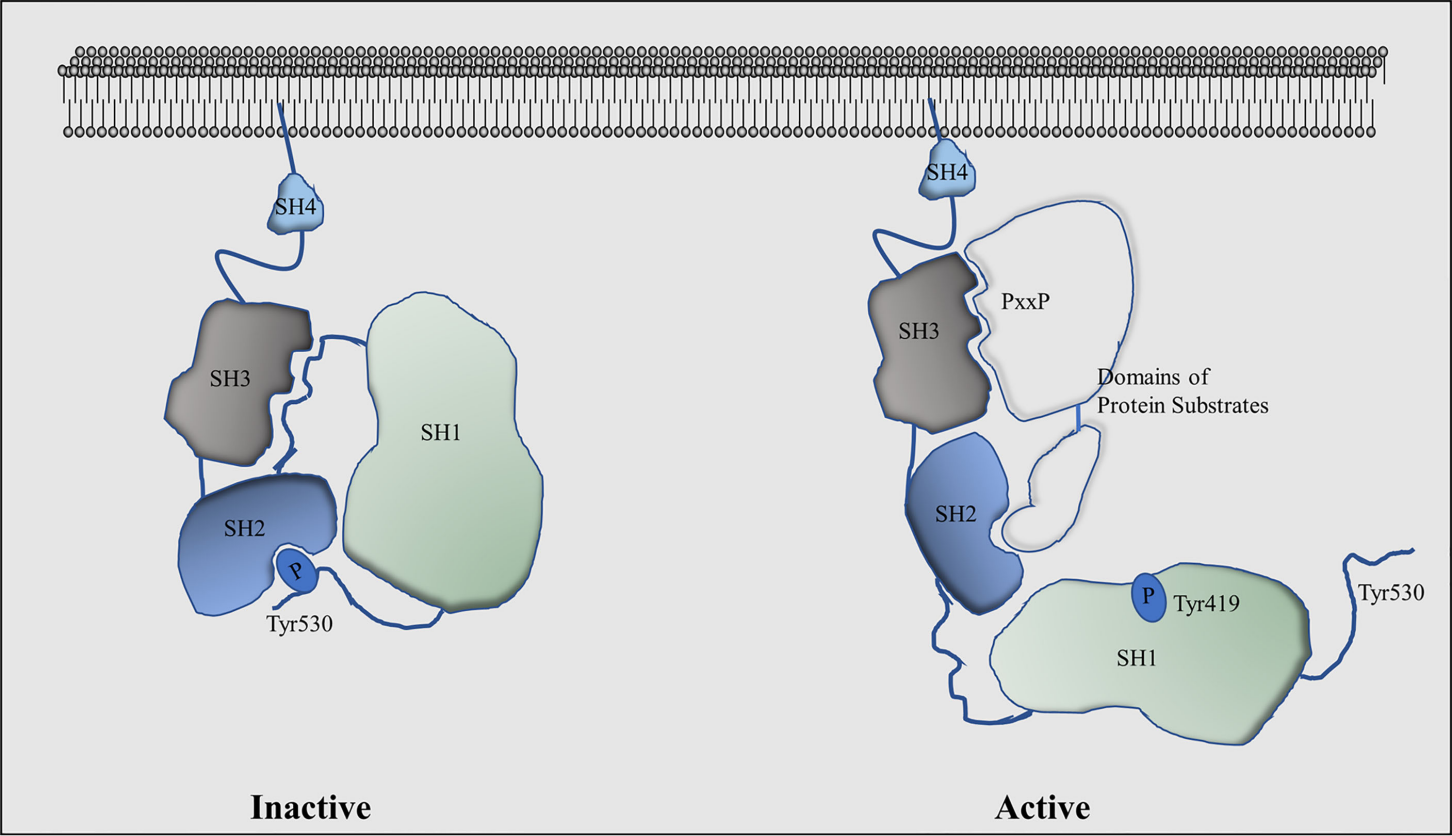
Functional domains of the Src kinase and their conformational changes in active and inactive stages.

Intracellular kinases play critical roles in regulating ligand-independent AR signalling, among which the proto-oncogene tyrosine kinase Src had been demonstrated to contribute to CRPC progression (59, 60). It is the founding member of the Src family of structurally-related protein tyrosine kinases that contains at least eleven members: Src, Yes, Fyn, Fgr, Blk, Hck, Lck, Lyn, Frk, Srm, and Brk (61). Src, Yes, and Fyn are widely expressed in many types of tissues, while Src is the most well-studied member. It has four functional domains (**Figure 2**): 1) the amino-terminal SH4 domain has myristoylation and palmitoylation post-translational modifications that are important for Src anchorage to the cell membrane, tyrosine kinase activity, and protein stability (62, 63); 2) the SH3 domain can be folded in a way that it interacts with the linker between the SH2 domain and the amino-terminal lobe of the SH1 domain to keep Src in an inactive conformation. The SH3 domain also recognizes the consensus sequence of PxxP as a minimal consensus target site within Src substrates (64). These PxxP motifs could be positioned in two opposite orientations defined by a positively charged residue (+xxPxxP or xPxxPx+) that interacts with a negatively charged SH3 peptide-binding surface (65, 66). However, there are several non-consensus SH3 targeting peptides have been reported (67), indicating that the SH3 domain could recognize atypical motifs that permit Src to interact with broader ranges of protein substrates to regulate complex intracellular signalling; 3) the SH2 domain has a conserved arginine residue that is required for the high affinity of the SH2 domain to interact with phospho-peptide (e.g., pYEEI) (68, 69). This domain forms intradomain interactions with the SH1 domain through the phosphor-Tyr530 to inactivate Src kinase activity (70); and 4) the SH1 tyrosine kinase domain, which contains the amino-terminal lobe that plays regulatory roles for Src kinase activity and the carboxyl-terminal lobe that has the intrinsic kinase activity (71). It contains a catalytic kinase domain which is activated by Tyr419 autophosphorylation and inactivated when Tyr530 is phosphorylated by C-terminal Src kinase (CSK).

Src activation is mainly controlled by its intramolecular phosphorylation modifications and by Src interacting proteins. Src is inactivated when Tyr530 is phosphorylated by CSK and its homolog CHK, and recognized by the SH2 domain resulting in a closed inactive Src conformation (72). In contrast, several protein phosphatases such as protein tyrosine phosphatase-α (PTPα), PTP1, SH2-containing phosphatase 1 (SHP1), and SHP2 can serve as activators of Src by dephosphorylating the Tyr530 (73–75). X-ray crystallography studies had revealed that the phospho-Tyr530 forms an intramolecular interaction with the SH2 domain. In conjunction with other intramolecular interactions between SH3 and SH1 domains, Src kinase activity is silenced. In contrast, phosphorylation of Tyr419 by either auto-phosphorylation or other kinases (76, 77) is critical for Src activation. This phosphorylation site is located in the “activation loop” that is conserved among other Src family members (78). When phosphorylated, it permits the substrate-binding pocket of the SH1 kinase domain to be exposed to allow the kinase to access its substrates (71), while mutation of this tyrosine will dramatically reduce Src activity. Another key factor to determine Src activity is the Src interacting proteins. While the SH2 and SH3 domains mediate intramolecular interactions with the SH1 kinase domain to keep Src in an inactivated state, peptides from Src interacting proteins could disrupt these intramolecular interactions to activate Src. It is now known that several tyrosine kinase receptors such as focal adhesion kinase (FAK) (79–81), epidermal growth factor receptor (EGFR) (82), HER2 (83), platelet-derived growth factor receptor (PDGFR) (84), and fibroblast growth factor receptor (85) can activate Src activity. Therefore, the activation of Src involves the selective recognization of SH2 and SH3 domains to their targeted ligand peptides in addition to SH1 recognization of its substrates, which model had been termed as ‘‘turned on by touch’’ mode (86).

Src was reported to play an important role in regulating cancer cell adhesion, migration, and invasion in addition to its established roles in cell proliferation. Src is required for the transition of the G2 to M phase of mammalian fibroblasts (87), as it forms a protein complex with p27 (88) and phosphorylates its Tyr88 and Tyr74 resulting in reduced p27 protein expression and subsequent cyclin-dependent kinases (CDKs) activation (89). Blocking Src by the PP2 inhibitor in lymphoma cells caused G2/M cell cycle arrest by suppressing the WEE1-CDK1 axis (90). Src inhibition by PD173955 induces mitosis arrest post chromosome condensation in the prophase phase but before the spindle assembly (91), which is consistent with the report showing that Src controls the G2 phase DNA damage checkpoint through activating ataxia telangiectasia mutated ATM, ATR, and Chk1 kinases (92). However, Src was shown to have more potent effects on cell adhesion and mobility in association with tumour metastasis. The cell-cell adhesion is formed by adherens junctions, desmosomes, tight junctions, and gap junctions et al. (93). Activated Src by protein tyrosine phosphatase 1B (PTP1B) can form a protein complex with the adherens junctions, which in turn promotes ubiquitination and subsequent endocytosis of E-cadherin for degradation (94). Activated Src can also stimulate the JNK signal pathway to regulate the expression of matrix metalloproteinases MMP2 and MMP9, and several tissue inhibitors of metalloproteinases (TIMPs) to break down ECM (95, 96). These Src functions together allow cancer cells to disassociate from adjacent cells and ECM and invade through ECM for metastatic dissemination.

## The Interplay Between Src and AR Signalling

**Figure 3:**
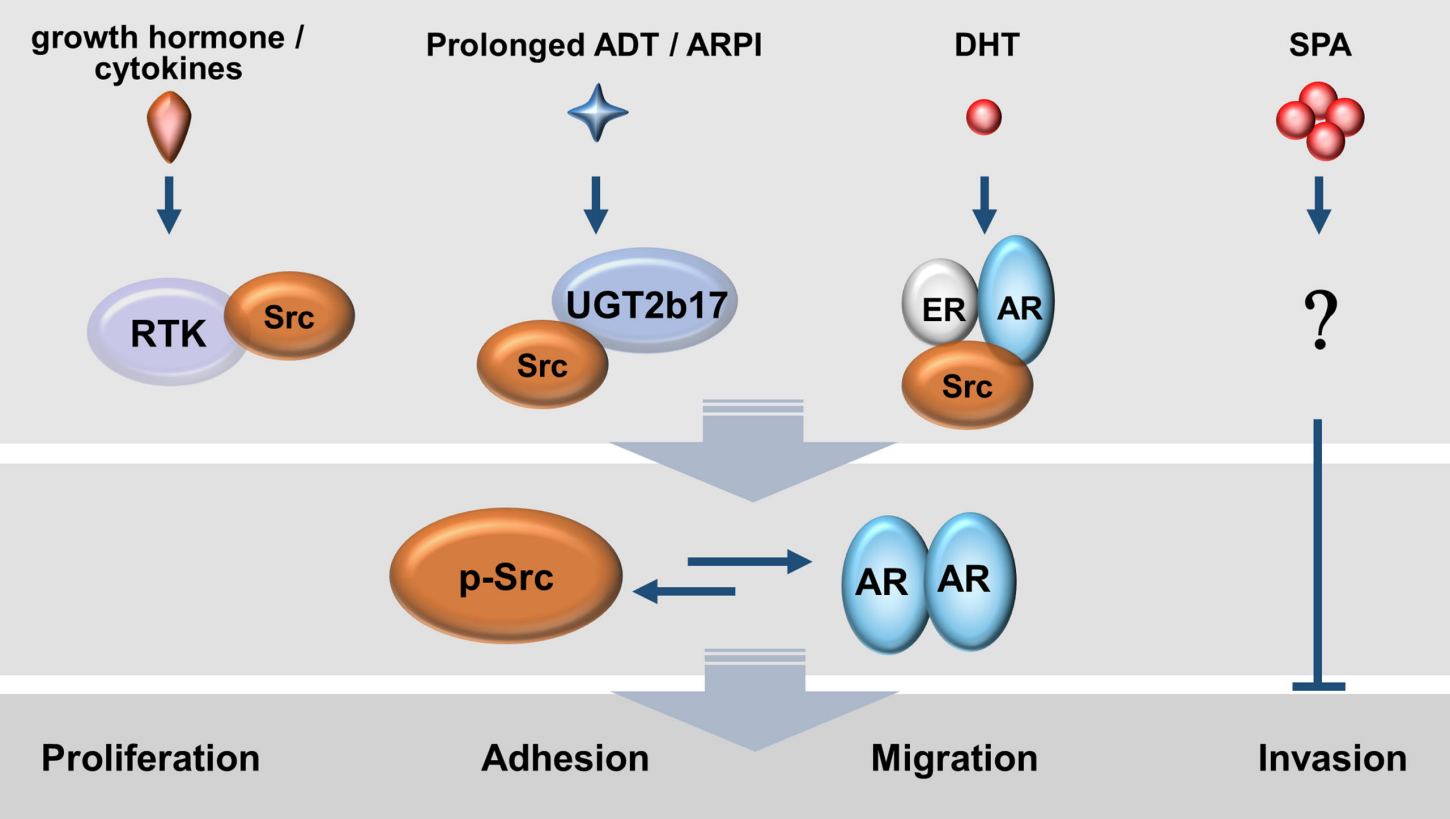
Mutual activations between AR and Src kinase exist in prostate cancer cells under various androgen conditions. Enriched growth factors and tumour-promoting cytokines as well as *de novo* androgen synthesis by tumour cells allow a persistent activation of the AR-Src axis.

The AR and Src can form a protein complex in PCa cells indicating that there exists crosstalk between both factors (97). Genetically engineered mouse models carrying overexpression of both AR and Src, but not alone, in normal prostate epithelial cells, can induce poorly or non-differentiated adenocarcinoma (98), supporting co-activation of AR and Src is required for prostate tumourigenesis. While AR gene amplification and overexpression are frequently observed in CRPC, upregulation of phospho-Src (Tyr419) and downregulation of CSK, an Src inhibitor, had also been reported (99, 100), indicating that active AR and Src signalling also co-exist during CRPC progression (**Figure 3**).

Mutual activation between AR and Src can exist not only under androgen depletion conditions but also in the presence of androgens. Under androgen deprivation conditions, growth factors (e.g., IGF-1 and EGF) and cytokines (e.g., IL-6 and IL-8) can mediate signalling to induce tyrosine phosphorylation of AR, and activate the transcriptional activity of AR (53, 58, 101). One key mediator of all these signal pathways is the Src (53, 54, 98). The mutant Src with inactive kinase activity downregulates AR transcriptional activity, while the constitutively active Src mutant (Y527F) induces AR nuclear translocation and AR activity in the absence of androgens (102). Blocking Src activities by small molecules inhibited ligand-independent, but not ligand-dependent AR activity and AR target gene expression (102, 103). Furthermore, prolonged androgen deprivation enhances UGT2b17 expression which in turn accelerates androgen clearance inside CRPC cells and at the same time promotes the formation of a UGT2b17-Src protein complex and subsequent Src activation (100). These findings indicate that growth factors, cytokines, and androgen catabolism enzymes can activate AR through Src in the absence of androgens.

In the presence of androgens, androgen-sensitive LNCaP cells were reported to stimulate Src autophosphorylation and its kinase activity by ligand-activated AR or by ligand-activated estrogen receptor beta (ERβ) (97, 104). The same results were replicated in breast cancer cells co-expressing AR and ER alpha (ERα) in the presence of estradiol. DHT or estradiol promotes a protein complex of AR/ER/Src. Src uses its SH2 domain to recognize the Tyr537 in ER, while the SH3 domain recognizes the PxxP motif in AR. In contrast, androgen deprivation abolishes Src autophosphorylation and kinase activity in LNCaP cells (97). This AR-activated Src activity, in turn, promotes PCa cell proliferation through accelerating G1-S phase transition (105), which effects can be blocked by Src inhibitors (104).

However, when androgen-sensitive PCa cell lines, as well as *ex vivo* PCa tissue, were challenged with supraphysiological doses of androgens (SPA), it induces cell cycle arrest at the G1 phase and cell senescence through cell cycling regulators such as CDK inhibitor p16, Rb1, and E2F1 (106). The SPA-induced cell cycle arrest can be partially alleviated by small molecule inhibitors against Src and Akt (106). These results suggest that Src has biphasic impacts on cell cycling depending on the levels of androgens exposed to PCa cells. It remains to be clarified how SPA-induced cell arrest is associated with Src activation.

Furthermore, Src can act independently of AR to stimulate PCa cell proliferation and migration. It stimulates AR negative PC3 and DU145 cell proliferation by accelerating the G1 phase of cell cycling (107). Inhibition of the Src activity by AZD0530 reduced the binding of β-catenin to cyclin D1 and c-Myc to block cell growth *in vitro*, and orthotopic DU145 xenograft growth *in vivo*. In addition, DU145 and PC3 cells have higher mobility activity than AR-positive LNCaP, which is correlated with high FAK/Src kinase activities. Treatment of PP2, the Src inhibitor, to both cell lines dramatically suppressed cell migration rates (108). The transgenic adenocarcinoma of mouse prostate (TRAMP) model is commonly used to study neuroendocrine prostate tumours since adenocarcinoma is initially developed in TRAMP mice and will commonly progress to neuroendocrine tumours (109). However, TRAMP mice with Src knockout have reduced adenocarcinoma establishment and metastasis, but have no impact on neuroendocrine tumour formation (110).

These studies indicate that Src can be activated by various mechanisms under different androgen regimes and exert biphasic actions on AR-positive PCa cell proliferation. Src also acts independently of AR signalling to regulate tumour cell proliferation and mobility. While multiple oncogenic pathways (e.g., aberrant AR, growth hormone, and tumour-promoting cytokines) all serve as the upstream regulator of Src activation, and enhanced Src activation is associated with CRPC progression. However, whether blocking Src activity will suppress CRPC remains to be defined. Src knockout mice were viable at birth but died due to impaired osteoclast function (111), suggesting that Src is not essential for benign cell viability. Transgenic mouse overexpressing Src alone in normal prostate epithelial cells did not induce malignancy (98), suggesting that Src itself is not critical for PCa development. Gain of overactive Src activity may be an adaptive response of PCa cells to survival under ARPI treatment. There may exist subpopulations of Src-dependent CRPC cells or a therapeutic window to co-target AR and Src during the early stage of tumour progression. In this case, the identification of biomarkers to stratify Src-dependent tumours will be critical to approving Src inhibitors to be used to treat patients.

## Src Inhibitors Tested in Prostate Cancer Patients

Results from preclinical studies using multiple PCa cells and xenograft models had built a reasonable rationale that targeting Src kinases would effectively suppress prostate tumour growth and progression in patients. Several phase I and II clinical trials in various tumour settings had also shown that dasatinib and saracatinib are well tolerated by patients with acceptable side effects, and the optimal dosing had been determined (112–115). Dasatinib had been approved by FDA to treat chronic myelogenous leukemia and acutely lymphoblastic leukemia (116). These results have encouraged the efforts to evaluate dasatinib in PCa patients (**Table 1**). Dasatinib had been tested in phase II trials as a monotherapy in chemotherapy naïve patients (114, 115), in which studies dasatinib was shown to be well tolerated with moderate treatment-related adverse effects. The most common treatment-related adverse events are fatigue, nausea and diarrhea (114, 115, 117–119). A further phase III READY clinical trial on 1522 PCa patients treated with docetaxel plus prednisolone with either placebo or dasatinib were performed (117). However, the results were disappointing since dasatinib did not show improved overall survival for chemotherapy naïve CRPC patients. Since Src had been demonstrated to contribute to tumours developing resistance to AR target therapy, a phase II trial was also designed to test 26 metastatic CRPC patients with no prior chemotherapy who received abiraterone plus prednisone together with placebo or dasatinib (118). Dasatinib did not show a significant improvement in progression-free survival. Another phase II trial has tested on abiraterone-resistant CRPC tumours treated with either dasatinib or sunitinib, an inhibitor targeting multiple receptor tyrosine kinases including VEGFR, Kit, and FLT-3 et al. (120). No difference in overall survival and time to treatment failure was observed between the dasatinib and sunitinib arms. These results raised doubts about the oncogenic property of Src during CRPC progression and urged more extensive characterization of the Src signalling under the context of heterogeneity of CRPC.

**Table 1:** Findings for clinical trials using Src inhibitor.

Patients	Src inhibitor	Number	Dose (daily)	OS	PFS	PSA response rate	Antitumor activity	Toxicity related to Src inhibitor	Reference
mCRPC	Dasatinib	48	100mg	Not available	24w (17%)	2.0%^A^	Yes^1,2^	Fatigue (43.8%) Nausea (27.1%) Diarrhea (27.1%)	PMID: 21539969
mCRPC	Dasatinib	47	100 or 70mg twice	Not available	24w (19%)	6.4%^A^	Yes^1,2^	Fatigue (44.7%) Nausea (46.8%) Diarrhea (61.7%)	PMID: 19920114
CRPC	Dasatinib	11	100mg	Not available	2.6m	Not available	No	Fatigue (54.0%) Nausea (82.0%) Diarrhea (67.0%)	PMID: 24788563
(treated with cediranib)	–	11	–	Not available	5.2m	Not available			
CRPC	AZD0530	28	175mg	Not available	8.0w	0.0%^B^	No^2^	Nausea (3.6%) Vomiting (3.6%) Lymphopenia (3.6%)	PMID: 19396016
CRPC	KX2-391	31	40mg twice	Not available	18.6w	10.0%^B^	No^1,2^	Fatigue (51.6%) LFT elevations (48.4%) Nausea (38.7%)	PMID: 23314737
mCRPC	Dasatinib	38	70mg twice	Not available	37.0d	27.0%^B^	No^1,2,3^	Fatigue (19.0%) Dyspnea (8.0%) Diarrhea (8.0%)	PMID: 23652277
mCRPC (treated with Abiraterone and prednisone)	Dasatinib	14	100mg	41.2m	15.7m	83.3%	No	Fatigue (71.4%) Anemia (57.1%) Diarrhea (42.9%)	PMID: 31227432
	–	12	–	26.9m	9.0m	100.0%			
				(p=0.4)	(p=0.2)	(p>0.05)			
mCRPC	Dasatinib	762	100mg	21.5m	11.8m	<79.0%	No	Diarrhea (56.0%) Fatigue (44.0%) Nausea (38.0%)	PMID: 24211163
(treated with Docetaxel)	Placebo	760	100mg	21.2m	11.1m	<84.0%			
				(p=0.9)	(p=0.2)	(p=0.1)			

CRPC, castration-resistance prostate cancer; mCRPC, metastatic castration-resistance prostate cancer; OS, overall survival; PFS, progression-free survival; PSA, prostate-specific antigen; m, months; w, weeks; d, days.

^A^PSA decline of ≥50% from baseline.

^B^PSA decline of ≥30% from baseline.

^1^Assessed using Prostate Cancer Working Group 2 (PCWG2) criteria.

^2^Assessed using Response Evaluation Criteria in Solid Tumors (RECIST) criteria.

^3^Assessed using Prostate Cancer Working Group 1 (PCWG1) criteria.

The inconsistent results from pre-clinical and clinical studies raised several questions. Is the Src activity critical for CRPC progression? Genetically engineered mouse model studies did not support that Src is essential for cell viability (98). The association of overactive Src with CRPC progression suggests that increased Src activity is an adaptive response of PCa cells for survival. However, it does not warrant that blocking Src will reduce PCa growth. Several other signallings such as PI3K/Akt or reprogrammed AR signalling may also be adopted by cancer cells. Src inhibition can force cancer cells to detour and use alternative survival mechanisms. In addition, pre-clinical studies using the PCa cell models and xenografts had shown that Src inhibitors induce cell cycle arrest and suppress tumour growth and cancer cell mobility. However, Src inhibitors may have limited capability to kill cancer cells to reduce tumour volumes. Whether they can cause PCa cell death and decrease xenograft volume needs further investigation.

Do all tumours/tumour cells respond to Src inhibitors? There exist inter-patient and intra-tumour heterogeneity of prostate tumours. Tumour cells carrying heterogeneous genetic and epigenetic backgrounds may respond to Src inhibitors differently. Particularly, prostate tumours in these clinical trials had progressed to the metastatic stage post castration therapies (117), previously receive radiation and chemotherapies (120), or had developed abiraterone resistance (120). The heterogeneity of these tumours is greater than that in treatment-naive tumours. Pre-clinical studies using xenografts derived from cell models may not capitulate the complete landscape of cancer cells in patients. Developing biomarkers that can be used to stratify Src-dependent tumours may help identify tumours sensitive to Src inhibition.

Are Src activities sufficiently blocked within patient tumours? Some clinical trials had used phosphor-Src protein (121) or mRNAs of CSF2, CD40L, GZMB, and IL-2 from peripheral blood cells (122) as surrogate biomarkers to confirm Src inhibition within tumours. However, the concentration of dasatinib that is sufficient to block Src in blood cells may not be sufficient for prostate tumour cells. As we discussed in previous sections, AR and AR-Vs could use their PxxP motifs to stimulate Src activity. Under prolonged androgen deprivation conditions, UGT2b17 is upregulated and forms a protein complex with Src, and stimulates its kinase activity. FAK is an upstream regulator of Src. FAK gene amplification is frequent in CRPC (123) and global phosphoproteomic profiling had found that overactivation of FAK kinase is a common mechanism by which PCa develops therapy resistance (124). These factors (e.g., AR, UGT2b17, and FAK) in PCa cells may be constitutively activating Src to counteract Src inhibitors. Neither did the phase II trials (114, 125) nor the phase III READY trial (117) successfully retrieve data confirming that Src is sufficiently inhibited by dasatinib inside the prostate tumours as discussed by the investigators. Together, there are several possibilities that Src inhibitors failed to show clinical benefits to PCa patients, and further investigations are needed.

## Conclusion

The Src signalling plays an important role to promote PCa development and CRPC progression. The interplay between Src and AR regulates PCa cell proliferation and metastasis under various androgen regimes, implying potential Src target therapy for CRPC. The negative results of Src inhibitors in clinical trials on PCa urge further mechanistic investigations at the molecular level on the roles of Src in PCa.

## Author Contributions

Writing-original draft preparation, LG and XD; writing-review and editing, BH and XD; All authors have read and agreed to the published version of the manuscript.

## Funding

This work was funded by Canadian Institute of Health Research operating grants (MOP-137007 & PJT-156150) to XD.

## Conflict of Interest

The authors declare that the research was conducted in the absence of any commercial or financial relationships that could be construed as a potential conflict of interest.

## Publisher’s Note

All claims expressed in this article are solely those of the authors and do not necessarily represent those of their affiliated organizations, or those of the publisher, the editors and the reviewers. Any product that may be evaluated in this article, or claim that may be made by its manufacturer, is not guaranteed or endorsed by the publisher.
